# Land surface temperature and transboundary air pollution: a case of Bangkok Metropolitan Region

**DOI:** 10.1038/s41598-024-61720-0

**Published:** 2024-05-13

**Authors:** Tanni Sarker, Peilei Fan, Joseph P. Messina, Ronald Macatangay, Pariwate Varnakovida, Jiquan Chen

**Affiliations:** 1 School of Planning, Design, and Construction and Center for Global Change and Earth Observations, Michigan State University, East Lansing, MI 48824 USA; 2 Department of Urban and Environmental Policy and Planning, Tufts University, 503 Boston Avenue, Medford, MA 02155 USA; 3 College of Arts and Sciences, University of Alabama, Tuscaloosa, AL 35487 USA; 4 Atmospheric Research Unit, National Astronomical Research Institute of Thailand, Chiang Mai, Thailand; 5 Department of Mathematics, Faculty of Science, King Mongkut’s University of Technology Thonburi (KMUTT), 126 Pracha-Uthit Road, Bang Mod, Thung Khru, Bangkok, 10140 Thailand; 6 Department of Geography, Environment, and Spatial Sciences and Center for Global Change and Earth Observations, Michigan State University, East Lansing, MI 48824 USA

**Keywords:** Urbanization, Air pollution, Remote sensing, Biomass burning, Transfer entropy, Environmental sciences, Environmental social sciences

## Abstract

In a rapidly urbanizing world, heavy air pollution and increasing surface temperature pose significant threats to human health and lives, especially in densely populated cities. In this study, we took an information theory perspective to investigate the causal relationship between diel land surface temperature (LST) and transboundary air pollution (TAP) from 2003 to 2020 in the Bangkok Metropolitan Region (BMR), which includes Bangkok Metropolis and its five adjacent provinces. We found an overall increasing trend of LST over the study region, with the mean daytime LST rising faster than nighttime LST. Evident seasonal variations showed high aerosol optical depth (AOD) loadings during the dry period and low loadings at the beginning of the rainy season. Our study revealed that TAP affected diel surface temperature in Bangkok Metropolis significantly. Causality tests show that air pollutants of two adjacent provinces west of Bangkok, i.e., Nakhon Pathom and Samut Sakhon, have a greater influence on the LST of Bangkok than other provinces. Also, the bidirectional relationship indicates that air pollution has a greater impact on daytime LST than nighttime LST. While LST has an insignificant influence on AOD during the daytime, it influences AOD significantly at night. Our study offers a new approach to understanding the causal impact of TAP and can help policymakers to identify the most relevant locations that cause pollution, leading to appropriate planning and management.

## Introduction

Against the backdrop of global warming, rising surface temperatures are now pervasive in the megacities of the Global South and are closely associated with low air quality^1,2^. While both heat and air pollution exposure are detrimental to human health, they become more severe when they act synergistically^3,4^. The scientific community has started to investigate the interaction mechanisms of these two environmental problems in urban area^5^. For example, Menon et al. reported that black carbon emissions exacerbated the urban heat islands (UHI) impact in China and India, warming the urban atmosphere and altering the stability and vertical movement of the planetary boundary layer^6^. Cao et al*.* found haze pollution to be a contributing factor to UHI that enhances nighttime surface UHI at 0.7 ± 0.3 K (mean ± 1 SE) for semi-arid cities across China^7^. Yang et al*.* found that in Beijing PM_2.5_ pollution affects UHI, leading to weakened UHI intensity in summer via aerosol-radiation interaction but strengthened UHI intensity during winter via aerosol-planetary boundary layer interactions^8^. In recent years, researchers have also investigated the spatial spillover linkages of air pollution in nearby areas of cities and have reported the spatial consequences of air pollution^9,10^. For instance, Jiang et al. examined the spatial and temporal patterns of air pollution in Beijing’s neighboring cities and used the Granger causality test to investigate how haze pollution in and around Beijing affected nearby cities^11^. Using a network framework, Zhang et al. analyzed the role of urbanization on the spillover of haze pollution in Cheng-Yu urban agglomerations in China^12^. Li et al*.* considered both urbanization and industrialization factors and their spillover effects on air pollutant emissions in Huang-Huai-Hai region of China^13^. A recent study by Nguyen et al*.* assessed the impact of pollution in Ho Chi Minh City (HCMC) in Vietnam and found that Indonesian biomass burning and Chinese haze are responsible for degrading HNMC’s air quality under certain meteorological conditions^14^. These studies, while providing meaningful insights into the regional spillover effects of pollution, disregarded the impact of transboundary air pollution on land surface temperature. Such an assessment of the contributions from local to distant sources can help cities take effective measures to reduce pollution loads and improve the thermal environment.

Thailand has been experiencing elevated land surface temperature (LST) along with year-round high levels of pollution originated from vehicular emissions, chemical and industrial activities, and traditional crop management, especially in Bangkok– the largest urban agglomeration^15,16^. Due to poor air quality, it is estimated that 29,000 people died prematurely in Thailand in 2021^17^. Air pollution also led to the loss of productivity of US $5.0–$6.4 billion in 2015 – 1.4–1.6% of the country’s total GDP^15^. Past studies have also reported the challenges to local and central governments in reducing and preventing transboundary air pollution (TAP) in Thailand^18–21^. Current studies highlight the nexus between air pollution and LST in Bangkok, albeit they are either confined to local urban areas or have solely concentrated on urbanization-related issues^22,23^. While the contribution of transboundary transport of air pollutants exclusively originating from biomass burning (24–38%) and non-biomass burning to total air pollution in Bangkok is substantial, no studies have quantitatively measured the contributions of TAP in affecting LST^18,24,25^. Clearly, there is a critical need for understanding the corresponding mechanisms so that better planning and management can occur.

**Figure 1:**
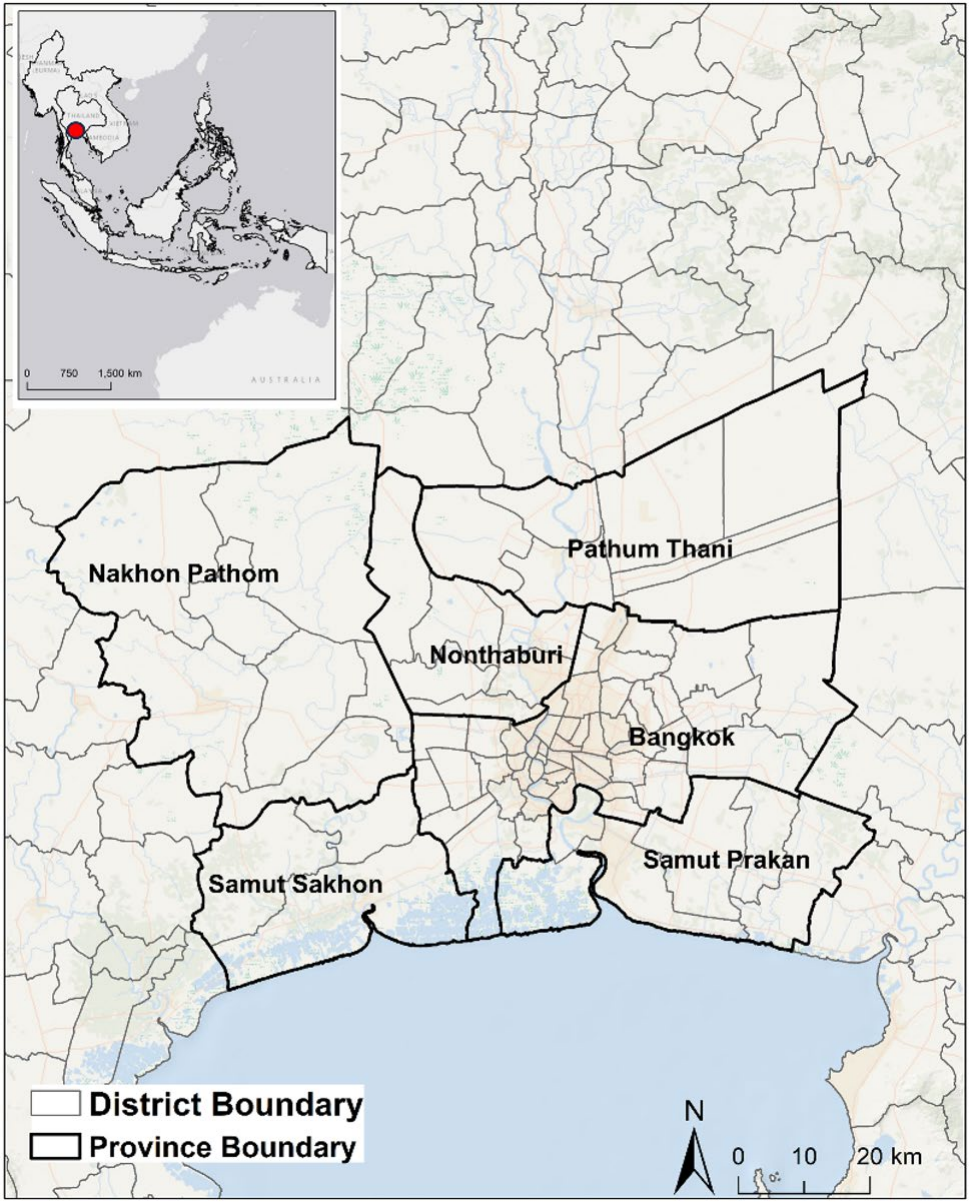
Study Area of Bangkok Metropolis Region (BMR), Thailand. *Note*: The map was generated by the authors by using ArcGIS v 10.4. Administrative division boundaries used in the map were downloaded from the Database of Global Administrative Areas (GADM) v 4.1 (https://gadm.org/data.html).

The main objective of this study is to use information theory to quantify the contribution of transboundary air pollution to the change of LST, using BMR as a case (Fig. 1). We hypothesize that air pollutants in Bangkok and its surrounding provinces contribute to the physical processes that drive LST in Bangkok and vice versa. We take an information theory perspective to analyze the interdependent dynamics of LST and air pollutants. We used transfer entropy (TE) of Information Theory in our causality analysis, as TE is a nonlinear generalization of the Granger causality test, model free, and accounts for both linear and nonlinear causal effects. The application of TE makes the attribution of a causal relationship between AOD and LST more reliable. The approach used in our study thus offers an innovative way to understand the causal impact of TAP and identify the most relevant locations that cause pollution and affect LST, leading to appropriate planning and management. This study can also provide valuable findings to policymakers in formulating regionally differentiated pollution control policies for Bangkok. Additionally, our study underlines that the effective management of environmental problems, such as LST and air pollution, which requires coordination between different administrative units due to the transboundary impacts revealed in this study.

## Data and method

### Study area

The Bangkok Metropolitan Region (BMR) covers Bangkok metropolis and five adjacent provinces, including Pathum Thani, Nonthaburi*,* Nakhon Pathom, Samut Sakhon, and Samut Prakan (Fig. 1). As the largest urban agglomeration in Thailand, BMR is inextricably intertwined in terms of transportation and economic growth^26^. Covering an area of 7756 km^2^, BMR is home to over 10 million people, with population density varying from approximately 700–19,500 people per km^2^. Most of the population is concentrated in the central city core along the banks of the Chao Phraya River. The urban area has expanded rapidly, creating a massive urban built-up area spreading from the city center to its surrounding provinces. In addition to pollution from traffic congestion, industrial production substantially contributes to total air pollution, as factories in and around Bangkok account for around 29% of the national total^25^. However, the total amount of pollutants varies due to changes in wind and weather patterns throughout the year. The study area has a tropical savanna climate under the Köppen climate classification and is under the influence of Asian monsoon. With the daytime temperature over 30 °C for most of the year, the area has three distinct seasons: a dry and hot season from February to May, a rainy season from June to October, and a cool season of November to January with relatively lower temperature. The simultaneous occurrences of rain and heat from June to October is typical of tropical monsoon regions. Nationally, pollutant concentrations decrease during the rainy season, while high air pollution episodes occur throughout the remaining seasons. Moreover, Bangkok suffers intense haze pollution from conventional management of crop residuals, especially during the dry season, with pollution also coming from surrounding regions. Around 54% of crop residues of rice and sugarcane are burned in the central region in Thailand annually^20^. Agricultural crop burning practices contribute significantly to seasonal spikes in pollution levels not only in locations practice such burning, such as Nakhon Pathom, but also in Bangkok where such burning is rarely practiced due to limited agricultural land.

### Data processing

We relied on remote sensing products from 2003 to 2020 to understand the process of TAP and LST interaction. We obtained daytime and nighttime land surface temperature at a 1 km resolution by utilizing the seamless gap-filled and clear sky, high spatiotemporal Moderate Resolution Imaging Spectroradiometer (MODIS) dataset^27^. This dataset overcomes the limitations of missing values due to clouds, shadows, and other atmospheric conditions. We converted per pixel LST from Kelvin to Celsius before aggregating to monthly minimum, mean and maximum daytime and nighttime LST over the study region. We used MODIS Aerosol (MCD19A2) V6 product at 1 km spatial resolution for quantifying Aerosol Optical Depth (AOD) as an indicator of air pollution. This dataset was derived by using the Multi-angle Implementation of Atmospheric Correction (MAIAC) algorithm, which is renowned for outperforming other available products^28,29^. Similar to LST data, we retrieved AOD products for the BMR region from 2003 to 2020 and converted to monthly averages for further analysis.

We analyzed angstrom exponent (AE) and single scattering albedo (SSA) over the BMR region to understand the aerosol composition. To measure AE, we utilized MODIS Deep Blue product for land (0.412–0.47 μm) and averaged at a monthly level for 2003–2020. This product has been particularly useful for bright surfaces, such as desert and urban areas and is not available over oceans^30^. Ozone Monitoring Instrument (OMI) OMAERUV v003 level-3 single scattering albedo data at 0.388 µm were retrieved monthly from (https://giovanni.gsfc.nasa.gov/giovanni/) for the period of 2004–2020^31^.

Land cover data at 30 m resolution was retrieved for 2000, 2010 and 2020 from GlobeLand30 product (http://www.globallandcover.com/home_en.html?type=data). We masked out water pixels to calculate LST since the high heat capacity of water could impact average diurnal LST. R software (version 4.0.2) was used for all analyses. We relied on the “RTransferEntropy” package to quantify directional information transfer between the systems and test whether they are statistically significant with *p* < 0.05^32^.

### Methods

Using BMR as a test site, we used transfer entropy to examine the causality between transboundary air pollution and LST (daytime and nighttime) during 2003–2020 and determined the directionality of the information exchange between different neighboring regions considering only statistically significant information transfers at *p* value < 0.05. Standard approaches like correlation coefficients fail to provide any insights about causality and are unable to infer which of the variables are driving the system. Commonly used metrics are unable to capture the full range of model behavior (e.g., nonlinearities, feedback loops, and emergent behaviors)^33,34^.

#### Information theory: transfer entropy

We applied information theory (IT), particularly the measurement of transfer entropy^35–37^, to quantify the contribution of air pollution to the change in land surface temperature in Bangkok. Information theory can be successfully employed to understand complex systems due to its advantage in interpreting causality^33–35^. It uses a probabilistic method to quantify the amount of information and uncertainty of information encoded in a time series. A key metric to quantify the information in IT is called entropy, which is a measure of the uncertainty associated with a random variable. According to Shannon^38^, entropy *H (x)* is defined as the number of bits to represent the amount of uncertainty (randomness) in a data source, and it can be expressed as

1 $$H\left(x\right)=-\sum_{i=1}^{n}\left[P\left(x\right) \cdot {\log }P\left(x\right)\right]$$

for a discrete random variable $x$, with possible outcome ${x}_{1}$,…….,${x}_{n}$, where $P\left(x\right)$ is the probability distribution associated with variable $x$. Similarly, the joint entropy of two discrete random variables x and y *H (x, y)* is simply the entropy of their pairing:$\left(x,y\right)$. This implies that if $x$ and $y$ are independent, then their joint entropy is the sum of their individual entropies.

2 $$H\left(x,y\right)= - {\sum }_{x\in x}{\sum }_{y\in y}\left[P\left(x,y\right)\cdot{\text{log}}\left[P\left(x,y\right)\right]\right]$$

The shared amount of information between *x* and *y* is called the mutual information (MI) $MI\left(x,y\right)$ and can be quantified as

3 $$MI\left( {x,y} \right) = \sum\nolimits_{{x \in x}} {\sum\nolimits_{{y \in y}} {\left[ {P\left( {x,y} \right) \cdot log\frac{{P\left( {x,y} \right)}}{{P\left( x \right) \cdot p\left( y \right)}}} \right]} }$$

where *MI* indicates how much information we gain about *x* after knowing *y*, and vice versa. Given its symmetric nature, *MI* can only provide the strength of association between two random variables; it is incapable of providing any information about the directionality of the couplings. In order to discern the directionality of couplings, Schreiber proposed transfer entropy (TE) based on the concept of Conditional Mutual Information^39^. Transfer entropy from a variable *x* to a variable *y* ${T}_{x \to y}$ can be defined as

4 $${T}_{x \to y}=\sum P{(y}_{t+1, }{y}_{t }^{n}, {x}_{t}^{m}) log\frac{P\left({y}_{t+1}|{y}_{t}^{n},{x}_{t}^{m}\right)}{P\left({y}_{t+1} |{y}_{t}^{n}\right)}$$

where ${y}_{n}^{t}$ = $({y}_{t, }{y}_{t-1}$,…….${y}_{t-n+1}$) and ${x}_{n}^{t}$ = $({x}_{t, }{x}_{t-1}$,…….${x}_{t-m+1}$) are the *n* and *m* orders of the Markov processes *x* and *y*. Transfer entropy from a process X to another process Y can be explained by the amount of uncertainty reduced in future values of Y by knowing the past values of X given the past values of Y. In this study ${TE}_{AOD \to LST}$ is the amount of information transfer from AOD of *x* city to LST of *y* city and can be interpreted by the amount of uncertainty reduced in future values of LST of *y* city by knowing the past values of AOD in *x* city given past values of LST of *y* city.

We computed pairwise TE between the variables at the monthly time scale using a 1-month lag time and presented them as a chord diagram. To avoid statistical artifacts, we implemented a randomized shuffled surrogate test and calculated the p-value. The study proceeded only when the value of information flux had a *p* value smaller than 0.05^40^.

#### SSA and AAE

Atmospheric aerosols can alter the total amount and distribution of solar radiation through absorption and scattering in the pollution layer and, therefore, contribute to climate change. The relationship between surface temperature and radiative forcing is strongly modulated by the interaction with aerosols^41,42^. SSA and AAE are two key parameters in determining the influence of aerosols on the earth’s radiative balance^43^.

Variation of SSA links to the strength of AOD in absorbing solar radiation and is modified with changes in aerosol content in the atmosphere. SSA values determine the cooling and warming outcome of the aerosol effect based on their structural and compositional characteristics. While high SSA values signify more scattering aerosols or a decrease in absorbing aerosols, lower values are linked to more absorbing aerosols^44^. Another parameter, AAE, a quantitative indicator of particle size, was analyzed to obtain information about the characteristics of AOD.

## Results

We found an increasing trend of LST over the study region, with the mean daytime LST rising faster than that of nighttime LST. Evident seasonal variations showed high aerosol optical depth (AOD) loadings during the dry period and low loadings at the beginning of the rainy season. Burning of crop residue, traffic congestion, and industrial activities have been identified as significant sources of air pollution in BMR, as indicated by Single Scattering Albedo (SSA) and Aerosol Angstrom Exponent (AAE). Our study revealed that TAP affected diel surface temperature in Bangkok Metropolis significantly. Causality tests show that air pollutants of two adjacent provinces west of Bangkok, i.e., Nakhon Pathom and Samut Sakhon, have a greater capacity in influencing the LST of Bangkok than other provinces. Also, the bidirectional relationship indicates that air pollution has a greater impact on daytime LST than nighttime LST. While LST has an insignificant influence on AOD during the daytime, it influences AOD significantly at night.

### Spatial and temporal variations of the LST during daytime and nighttime

**Figure 2:**
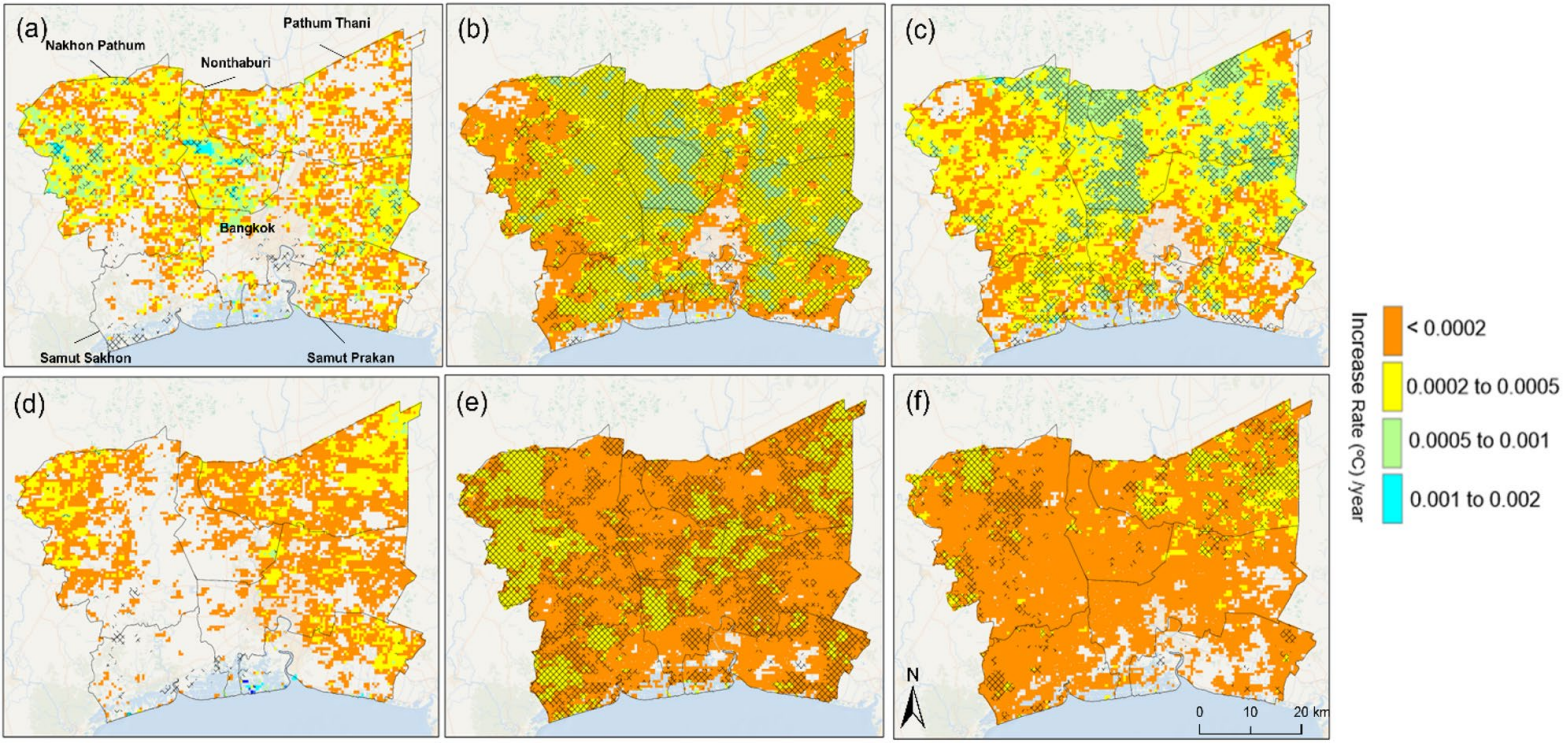
The trending of diel land surface temperature (LST) over the years of 2003–2020 (**a**) minimum daytime LST, (**b**) mean daytime LST (**c**) maximum daytime LST and (**d**) minimum nighttime LST, (**e**) mean nighttime LST and (**f**) maximum nighttime LST. *Note*: Hatched regions indicate statistically significant (*p* < 0.05) trends (increase or decrease of the temperature). The analysis was conducted, and the maps were generated by the authors by using R v 4.0.2 and ArcGIS v 10.4. Administrative division boundaries used in the maps were downloaded from GADM v 4.1 (https://gadm.org/data.html).

During the study period of 2003–2020, the mean *daytime LST* increased at a higher rate than the mean *nighttime LST* around BMR (Fig. 2). The spatial–temporal trend was computed by a linear fit to observe how the average, minimum and maximum *nighttime LST* and *daytime LST* changed over space for 18 years in and around BMR.

We found that from 2003 to 2020, ~ 63% of the area of BMR experienced a significant increase in mean *daytime LST*, whereas ~ 60% had a significant increase in mean *nighttime LST*. Within BMR, the Bangkok metropolis had ~ 66% and ~ 58% of its area experienced significant increases in mean *daytime LST* and mean *nighttime LST*, respectively. Similarly, significant increases in mean *daytime LST* and *nighttime LST were* found in ~ 95% and ~ 55% of the area of Nonthaburi, respectively, and ~ 70% and ~ 61% of the area of Pathum Thani, respectively*.* More areas of Nakhon Pathom province experienced a significant increase in mean *nighttime LST* (~ 70% of the area) than in mean *daytime LST* (~ 65% of the area). Noticeably, the minimum *daytime LST* increased significantly only in a small part of Nakhon Pathom (~ 6% of the area) and the northeastern part of Bangkok metropolis (~ 5% of the area).

Nonthaburi Province had a significant linear warming trend with an annual increase in mean *daytime LST* of 0.17 °C/y during the study period, well above that of other provinces in Bangkok metropolis (0.10 °C /y), Pathum Thani (0.09 °C/y), Samut Sakhon (0.07 °C/y), Nakhon Pathom (0.06 °C/y), and Samut Prakan (0.06 °C /y). In contrast, the annual increase in mean *nighttime LST* appeared similar for all provinces, with Nakhon Pathom having a slightly higher annual increase (0.06 °C /y) than others (0.05 °C/y). It should be noted that the minimum *daytime LST* in Nakhon Pathom increased at an annual rate of 0.08 °C/y, surpassing the annual change rate of the mean (0.06 °C/y) and maximum (0.06 °C/y) *daytime LST* of BMR. A countertrend, though modest, was identified in Bang Kachao Park – an urban forest park, experienced a cooling trend with an annual change of mean *daytime LST* of − 0.001 °C/y.

### Aerosol loading (AOD) distribution

**Figure 3:**
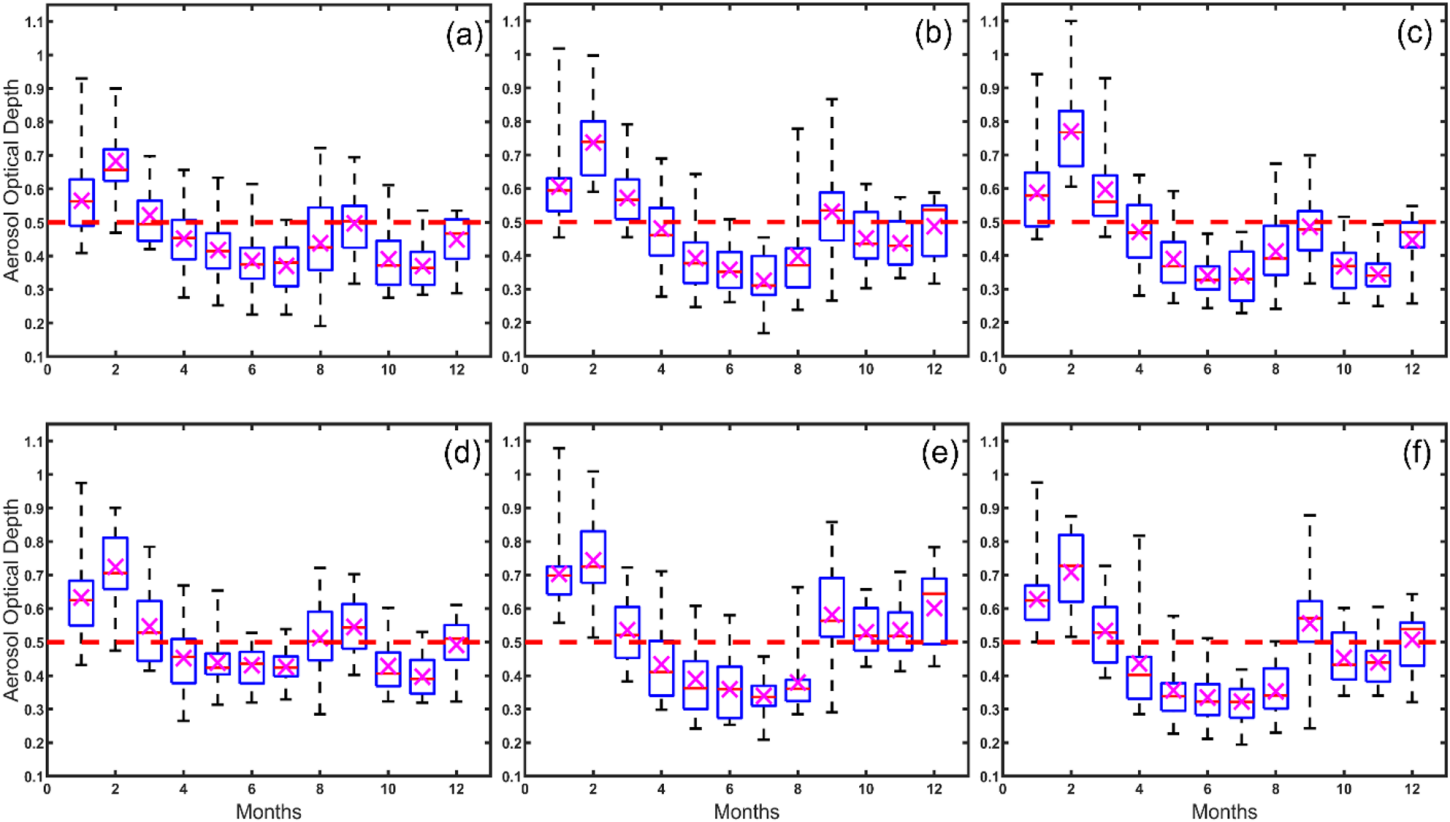
Intra-annual variation of AOD for 2003–2020 (**a**) Bangkok metropolis, (**b**) Nonthaburi (**c**) Pathum Thani (**d**) Samut Prakan (**e**) Samut Sakhon and (**f**) Nakhon Pathom. *Note*: Magenta cross ‘x’ indicates mean value and magenta solid line ‘–’ represents median value in the box.

Intra-annually, the AOD loading showed significant seasonal differences (Table 1). The average AOD concentration was high during the dry season (February–May) and low in the cool (November–January) and rainy seasons (June–October), except for Nakhon Pathom and Samut Sakhon, where the average AOD concentration was much higher during the cool season. The average AOD concentration was particularly high during January and February and low in July for all provinces (Fig. 3a–f). Over the 18 years, the average AOD values in February in Bangkok, Nakhon Pathom, Nonthaburi, Pathum Thani, Samut Prakan, and Samut Sakhon were 0.68 ± 0.10, 0.70 ± 0.03, 0.73 ± 0.03, 0.77 ± 0.003, 0.72 ± 0.04 and 0.74 ± 0.01, respectively. The Maximum AOD values showed a similar seasonal change to the average AOD values. It is worth noting that AOD loading started to decrease in April for all provinces.

**Table 1:** AOD loading in different seasons during 2003–2020 in BMR.

Season	Province	Average ± SE	Dry	Rainy	Cool
Dry	Bangkok Metropolis	0.519 ± 0.06	1.000	–	–
Rainy		0.416 ± 0.048	0.000****	1.000	–
Cool		0.461 ± 0.068	0.005 ***	0.0272 *	1.000
Dry	Nakhon Pathom	0.508 ± 0.064	1.000	–	–
Rainy		0.404 ± 0.048	0.000***	1.000	–
Cool		0.525 ± 0.068	0.425	0.000***	1.000
Dry	Nonthaburi	0.545 ± 0.058	1.000	–	–
Rainy		0.412 ± 0.052	0.000***	1.000	–
Cool		0.510 ± 0.072	0.0875	0.000***	1.000
Dry	Pathum Thani	0.557 ± 0.063	1.000	–	–
Rainy		0.389 ± 0.042	0.000***	1.000	–
Cool		0.459 ± 0.063	0.000***	0.000***	1.000
Dry	Samut Prakan	0.540 ± 0.066	1.000	–	–
Rainy		0.468 ± 0.035	0.000 ***	1.000	–
Cool		0.507 ± 0.067	0.0906	0.0506	1.000
Dry	Samut Sakhon	0.526 ± 0.076	1.000	–	–
Rainy		0.438 ± 0.053	0.000 ***	1.000	–
Cool		0.613 ± 0.078	0.000 ***	0.000 ***	1.000

***P < 0.001.

Dry Seasons (Feb–May) had high AOD concentrations, while cool (Nov–Jan) and rainy seasons (Jun–Oct) had low concentrations. However, Nakhon Pathom and Samut Sakhon had higher concentrations during cool seasons.

### Causality between surface temperature and aerosol intensity

**Figure 4:**
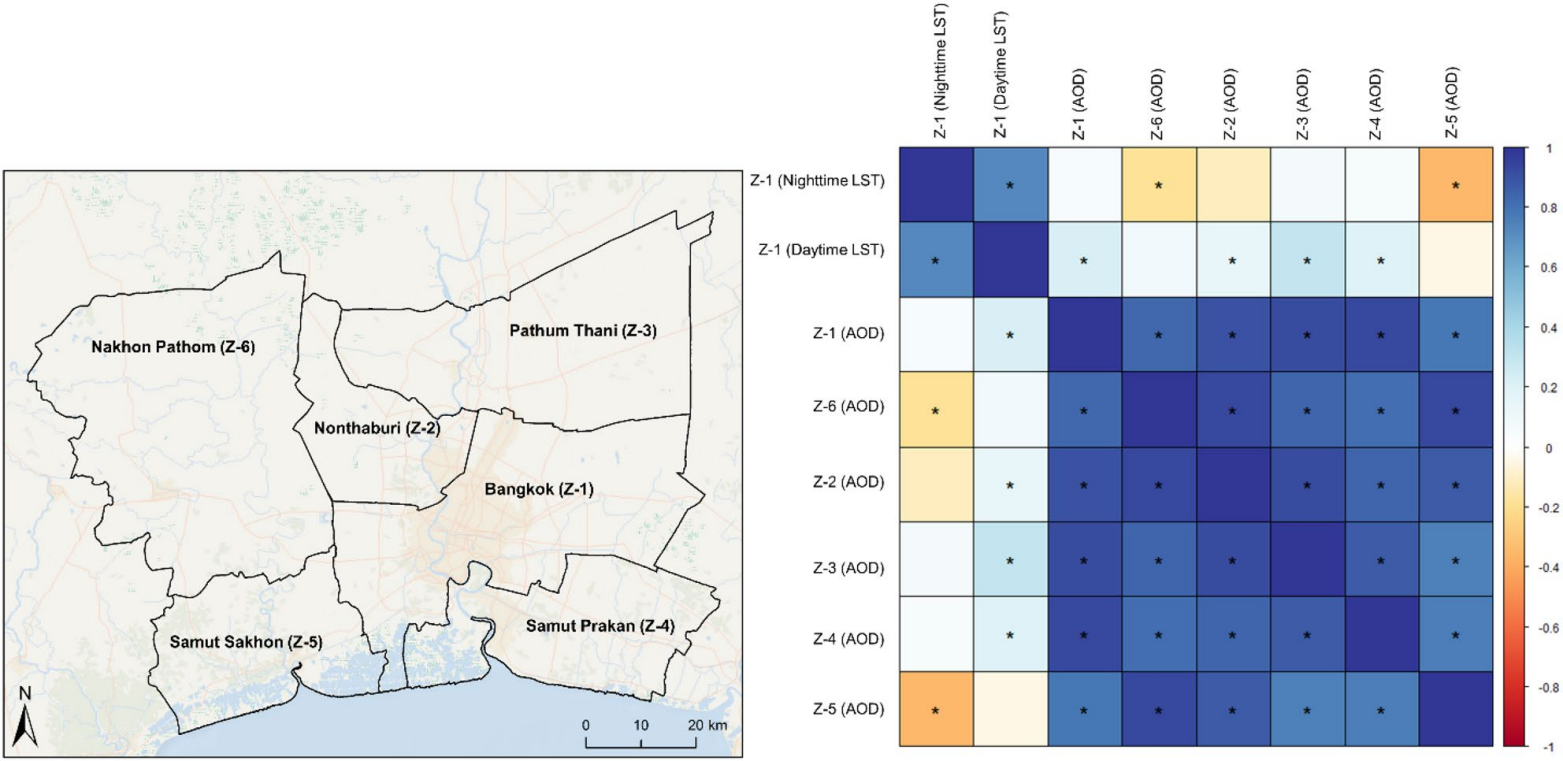
The heat map of the Pearson correlation coefficient of AOD loadings of each province and with LST (daytime and nighttime) of BMR.

The Pearson correlation coefficient test was performed and presented as a heat map in Fig. 4 to demonstrate the interdependencies between the time series of AOD for each province (Bangkok, Nonthaburi, Pathum Thani, Samut Prakan, Samut Sakhon, and Nakhon Pathom are hereafter referred as Z-1, Z-2, Z-3, Z-4, Z-5, Z-6, respectively) along with their association with the Bangkok LST time series. The correlation coefficients (r) between each pair of AOD time series are positive and significant. However, the association of Bangkok LST for both daytime and nighttime with other provinces’ AOD is mixed. Z-1 (*nighttime LST*) was negatively correlated with AOD of Z-2 (r = − 0.12, *p* > *0.05*), Z-5 (r = − 0.35, *p* < *0.05*), and Z-6 (r = − 0.19, *p* < *0.05*) and positively with AOD of Z-1 (r = 0.05, *p* < *0.05*), Z-3 (r = 0.07, *p* > *0.05*), and Z-4 (r = 0.05, *p* > *0.05*). On the other hand, Z-1 (*daytime LST*) was positively correlated with AOD of Z-1 (r = 0.23, *p* < *0.05*), Z-2 (r = 0.14, *p* < *0.05*), Z-3 (r = 0.3, *p* < *0.05*), Z-4 (r = 0.2, *p* < *0.05*), and Z6 (r = 0.09, *p* > *0.05*) and negatively associated with Z-5 (r = − 0.04, *p* > *0.05*).

**Figure 5:**
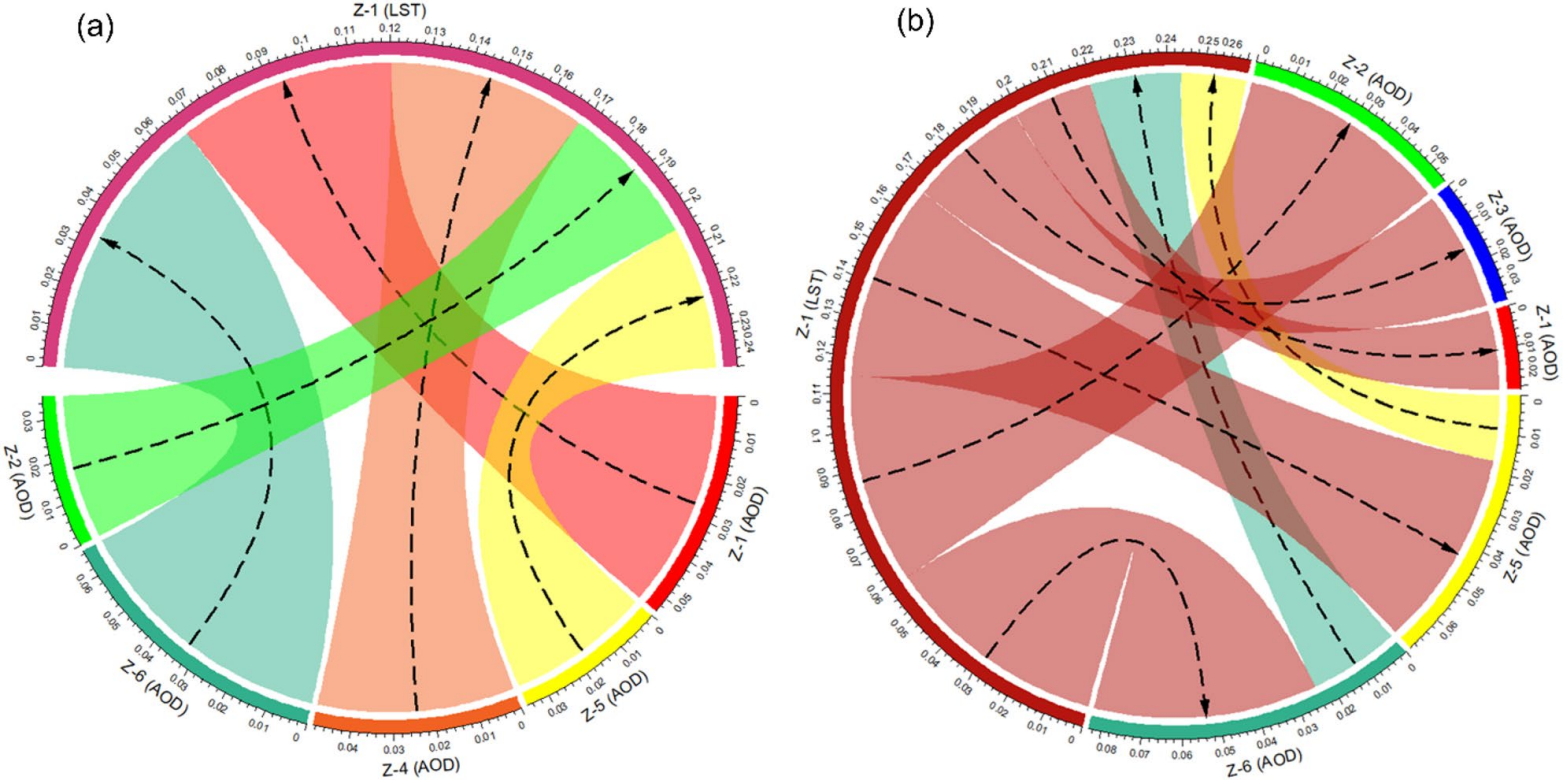
Chord diagram showing statistically significant transfer entropy between LST and AOD during 2003–2020 (**a**) daytime (left) and (**b**) nighttime (right).

Directional information flows among provinces, namely AOD of Z-1, Z-2, Z-3, Z-4, Z-5, and Z-6 to diurnal LST of Z-1 and vice versa revealed a much higher proportion of the information transfer going to Z-1 *daytime LST* with an emphasis on AOD from Z-6, Z-1, Z-4, Z-5, and Z-2 as contributing factors, respectively (Fig. 5a). The information transfer to Z-1 *nighttime LST* was much smaller than to Z-1 *daytime LST*, and AOD from Z-5 and Z-6 act as contributing factors (Fig. 5b). In comparing the TE between other provinces, information transfer of AOD from Z-6 was comparatively higher for both day and night LST in Z-1. One interesting difference in these chord diagrams was the lack of information transferred to AOD in all provinces from Z-1 *daytime LST*, while AOD of all provinces received information from Z-1 *nighttime LST*. The plausible explanation for this asymmetric influence of AOD pollution on *daytime LST* and *nighttime LST* could be due to the fact that the relationship between LST and radiative forcing is strongly modulated by the interaction with aerosols^41,42^. It indicates that air pollution has a greater impact on the land surface temperature during the day than at night.

### Mechanism of single scattering albedo and aerosol angstrom exponent

**Figure 6:**
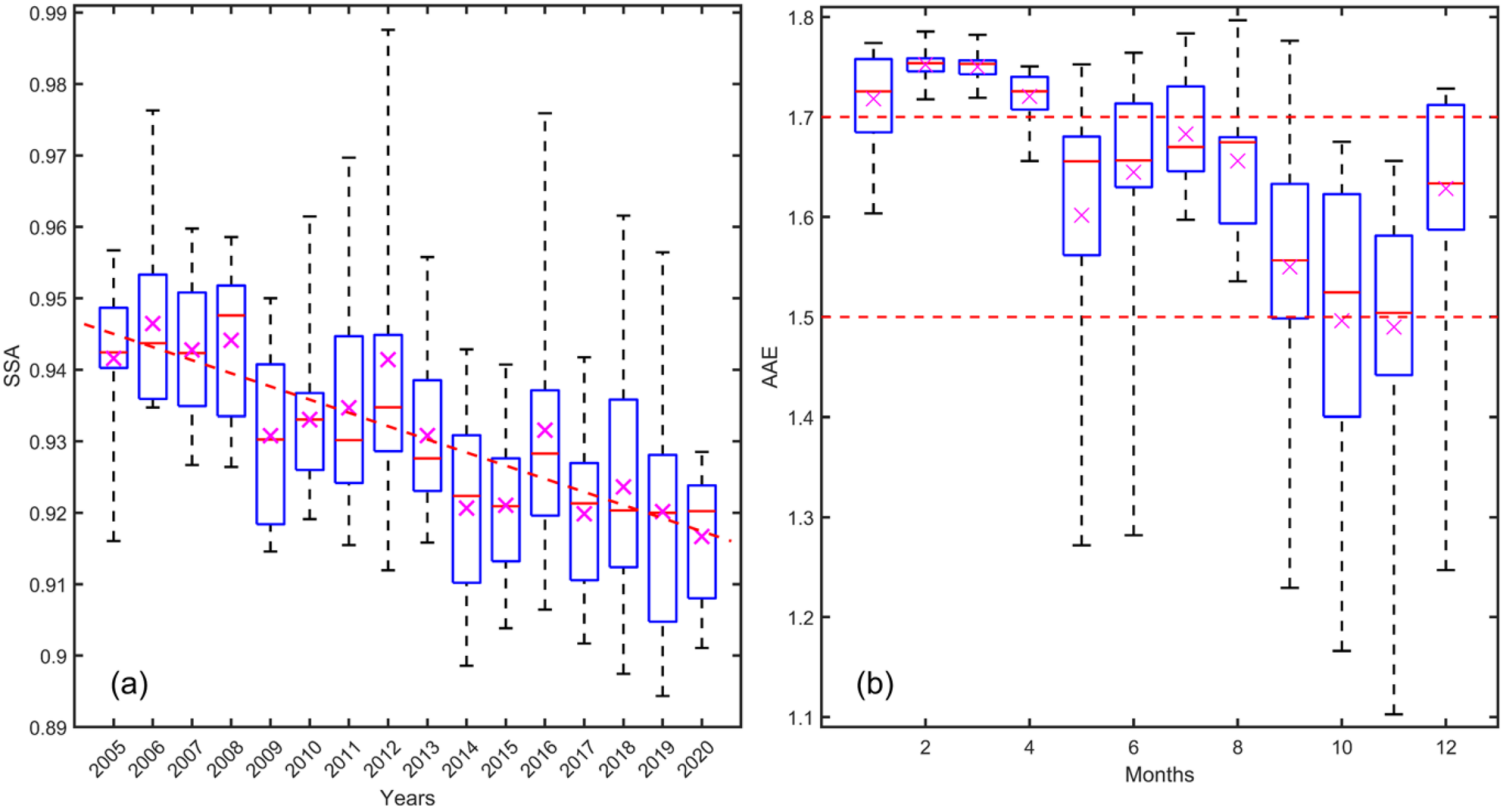
(**a**) Inter-annual variation of Single Scattering Albedo (SSA) over BMR during 2004–2020 and (**b**) intra-annual variation of Aerosol Angstrom Exponent (AAE) over BMR during 2003–2020. Note: Magenta cross ‘x’ indicates mean value whereas red line ‘–’ represents median value in the box.

Intra-annually, the average SSA experienced a decreasing trend of 0.0018/year (R^2^ = 0.78) over BMR during 2004–2020, suggesting that aerosols have become more absorbing (Fig. 6a). The inter-annual variation of AE for the years 2003–2020 ranged from 1.489 to 1.752 (Fig. 6b). The highest and lowest average AE values were observed as 1.752 ± 0.014 and 1.4961 ± 0.142 in February and November, respectively. High AE values indicate the dominance of fine particles, whereas low AE values suggest that most aerosols are coarse particles resulting from biomass burning during the dry season.

## Discussion

### LST patterns and information transfers between LST and AOD

The spatial variation of LST for both daytime and nighttime across BMR during 2003–2020 was reliably depicted by the linear fit. We found some noteworthy changes in LST over BMR. While the minimum daytime temperature (0.08 °C/y) rose at a higher rate than that of the mean (0.06 °C/y) and maximum daytime temperature in Nakhon Pathom (0.06 °C/y), the minimum *daytime LST* increased significantly only in some part of Nakhon Pathom (~ 6% of area) and the northeastern part of Bangkok metropolis (~ 5% of area). It should also be noted that in areas primarily characterized by urban green and blue spaces, there was a declining trend in the mean *daytime LST* (− 0.001 oC/y), further confirming the contribution of urban green/blue space in mitigating the impact of urban heat island^22,23^. The cooling trend is identified in the natural land surface environment near Bang Kachao – a tropical urban forest park with an area of 20 km^2^ located in the Chao Phraya lower floodplain. Also, some places in BMR and vicinities warmed up faster during daytime than nighttime; this disproportionate warming could be attributed to latent heat flux caused by differences in the water table and soil moisture^45,46^. Our findings on seasonal differences in AOD loadings over BMR may be mainly ascribed to meteorological conditions and pollution sources. During the rainy season, the pollution concentration is lower due to the favorable atmospheric dispersion conditions caused by monsoon winds and low air pressure. In contrast, during the dry and cool seasons, high air pollution episodes are ubiquitous, likely driven by weaker winds and air pressure inversions near the ground, as reported in previous studies^15^.

While bidirectional information transfers exist between LST and AOD during nighttime and daytime, the directionality of AOD towards *daytime LST* is significantly (*p* < *0.05*) stronger than the perturbation caused by *daytime LST* to AOD. In contrast, at night, *LST → AOD* is stronger than *AOD → LST*. This may be due to the dynamic relationship between LST and AOD. From a climatic point of view, a change in tropospheric aerosol concentrations results in strong heating or cooling effects^41^. On one hand, high LST could encourage the upward transport of aerosol particles, leading to transboundary pollution. On the other hand, while heating from land surface temperature can carry pollutants to the top of the boundary layer through vertical advection, it is only effective up to a certain altitude. Eventually, the concentration of pollution aloft will be so high that diffusion will increase the concentration of pollutants near ground level. Additionally, we find that the air pollutants coincident of Nakhon Pathom and Samut Sakhon have a greater capacity to influence the LST of Bangkok metropolis than other neighboring provinces.

We confirm that aerosol absorption leads to a positive radiation balance anomaly, affecting radiative heating of the aerosol layer^44,47^. SSA, a parameter quantifying scatter/absorption of solar radiation by aerosols, had a decreasing trend over BMR at a rate of 0.0018/year (R^2^ = 0.78), indicating aerosols comprising the column are more absorbent and mainly attributed to the prevalence of black carbons from urban pollution that is emitted from combustion sources and agricultural burning in and around the BMR. This result is consistent with a recent study of Li et al*.* who found relatively low SSA values over Southeast Asia where biomass burning related black carbon and organic carbon is intense^44^. For the months of January, February, and March, we observed AE of > 1.7, indicating the dominance of fine-sized anthropogenic aerosols, which arise mostly from the combustion of fossil fuels and biomass burning^48–50^. The two parameters of SSA and AAE together provide an understanding of the sources and properties of aerosol loadings over BMR.

### Urban and agricultural land use changes in Bangkok Metropolis and surroundings

**Figure 7:**
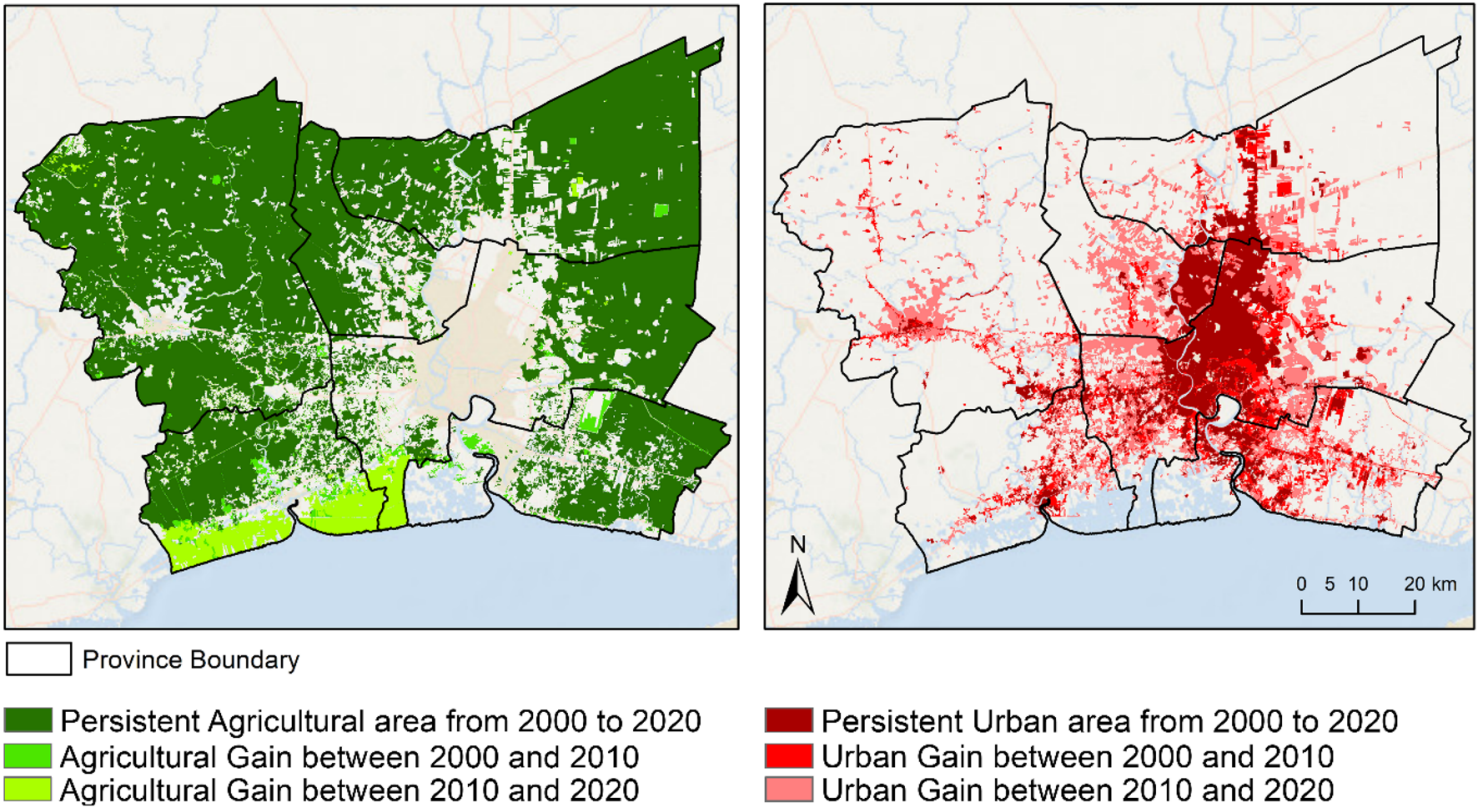
Urban and agriculture gains and persistence in Bangkok metropolis and five surrounding provinces during 2000–2020. *Note* The maps were generated by the authors by using ArcGIS v 10.4 and administrative division boundaries used in the maps were downloaded from GADM v 4.1 (https://gadm.org/data.html).

Our findings on LST associated with urban and agricultural land use changes in recent decades in BMR and its surrounding areas, as LST is primarily influenced by surface type, with different land cover compositions playing significant roles^51,52^. Urban expansion in our study region, facilitated significantly by the expansion of transportation infrastructure, including the construction of highways and railways, extended beyond administrative boundaries, encroaching into the surrounding agricultural areas. Urban built-up areas expansion is characterized by (1) mainly edge growth from existing main urban center of Bangkok, (2) spill-over the administrative boundaries of provinces, particularly towards west and east, and (3) further expansion of center cities of all five surrounding provinces (Fig. 7). This expansion has led to the development of functional units within different districts, with the inner city (Bangkok) becoming increasingly institutional and commercial, while the surrounding provinces urban expansion has been characterized by predominantly residential and industrial zones^53^ (Fig. 7). The rise in minimum daytime temperature in the inner city of Bangkok indicates a strong influence of urban morphology in shaping LST patterns. This finding aligns well with previous research indicating that the densely built-up areas influence the LST of Bangkok^54,55^. Furthermore, the province of Nakhon Pathom, located to the west of Bangkok, experiences severe air pollution attributed to a combination of vehicular emissions and emissions from numerous coal-powered factories, exacerbated by urban topography characterized by high-rise buildings impeding wind dispersion. Meanwhile, the modulation of land surface temperature is significantly influenced by the onsite activities of agricultural fields throughout the year. Agricultural land still dominates the surrounding provinces of Pathum Thani, Nonthaburi*,* Nakhon Pathom, Samut Sakhon, and Samut Prakan, with aquaculture characterized the agricultural land gain of 2010–2020 in the coastal area of Samut Sakhon and Bangkok metropolis (Fig. 7). The minimum daytime temperature is rising in rural zones in the western part of the Nakhon Pathom, where rice crop fields dominate and play an important role in assuring BMR’s food security. The agricultural lands, which were converted into bare soil after harvesting, often contain high organic matter with high heat capacity, thus influencing the heating and cooling process of the land surface thermal environment^54,56,57^.

### Limitations and future studies

Our current study does have some limitations that should be addressed in the future. First, as our study area focuses on five provinces that share common borders with Bangkok metropolis, we did not consider the influence of the region beyond the study area. Nevertheless, other regions (e.g., central, north, and northeast) of Thailand, as well neighboring countries, may have significant influences on the air quality of BMR. For example, previous studies have reported that long-range transport of biomass burning emissions from neighboring countries, such as Myanmar, Laos, and Vietnam affect the air quality of Thailand^14^. Second, despite AOD serving as a reliable indicator of air pollution, AOD has an inherent limitation as it exclusively focuses on particulate matter and does not encompass gaseous pollutants such as ozone and nitrogen dioxide. Future studies should consider incorporating other pollutants. Third, in addition to air pollutants, meteorological factors, such as temperature, cloudiness, humidity, the frequency and intensity of precipitation, and wind patterns, together with geographic features of landscapes, can individually and synergistically drive air quality^58^. For example, lower LST due to aerosols may have influenced the aerosol-planetary boundary layer (PBL) heights over Bangkok causing air stagnation that makes pollutants difficult to disperse, as has been shown for Chiang Mai^59^. Furthermore, mega cities in China have been found to have enhanced air pollution due to PBL interactions^60^. Nevertheless, we have not considered these factors in our current study. In future studies, we should incorporate meteorological and environmental factors toward understanding the causal relationship between land surface temperature and air pollutants. In addition, regional climate models (e.g., Weather Research and Forecasting model coupled with Chemistry (WRF-Chem)) could be utilized to better understand the spatial temporal distribution of pollutants and impacts on regional meteorology during biomass burning or forest fire events^61^. Fourth, our study spanned the period from 2003 to 2020, with a monthly time interval, resulting in a total of 204 samples. In future studies, we could improve the sample size to further elucidate the causal relationship between air pollution and land surface temperature.

## Conclusion

This study uses an information theory perspective to investigate the causal relationship between diel land surface temperature (LST) and transboundary air pollution (TAP) from 2003 to 2020 in the Bangkok Metropolitan Region (BMR), which includes Bangkok Metropolis and its five adjacent provinces. We found that the study region had an overall increasing trend of LST, with the mean daytime LST rising faster than nighttime LST. While the dry season had high aerosol optical depth (AOD) loadings, low loadings began as the rainy season started. TAP affected diel surface temperature in Bangkok Metropolis significantly with causality tests indicating that air pollutants of two adjacent provinces west of Bangkok, i.e., Nakhon Pathom and Samut Sakhon, have a greater influence on the LST of Bangkok than other provinces. Air pollution has a greater impact on daytime LST than nighttime LST. Furthermore, while LST has an insignificant influence on AOD during the daytime, it influences AOD significantly at night. Our study offers a new approach to understanding the causal impact of TAP and can help policymakers to identify the most relevant locations that cause pollution, leading to appropriate planning and management.

## Data Availability

Data used in this study are collected from the following sources: Land cover data are from GlobeLand30 (http://www.globallandcover.com/home_en.html?type=data); Angstrom exponent and single scattering albedo dataset are from (https://giovanni.gsfc.nasa.gov/giovanni/); Aerosol Optical Depth data are from the MODIS Aerosol product data Archive in Google Earth Engine (https://earthengine.google.com).
